# A Connexin Gene (*GJB3*) Mutation in a Chinese Family With Erythrokeratodermia Variabilis, Ichthyosis and Nonsyndromic Hearing Loss: Case Report and Mutations Update

**DOI:** 10.3389/fgene.2022.797124

**Published:** 2022-05-23

**Authors:** Yajuan Gao, Qianli Zhang, Shiyu Zhang, Lu Yang, Yaping Liu, Yuehua Liu, Tao Wang

**Affiliations:** ^1^ State Key Laboratory of Complex Severe and Rare Diseases, Department of Dermatology, Peking Union Medical College Hospital, Chinese Academy of Medical Science and Peking Union Medical College, National Clinical Research Center for Dermatologic and Immunologic Diseases, Beijing, China; ^2^ Chinese Academy of Medical Sciences and Peking Union Medical College, Beijing, China; ^3^ Department of Medical Genetics and National Laboratory of Medical Molecular Biology, Institute of Basic Medical Sciences, Chinese Academy of Medical Sciences and Peking Union Medical College, Beijing, China

**Keywords:** connexin gene, GJB3, erythrokeratodermia variabilis, ichthyosis, nonsyndromic hearing loss

## Abstract

**Background:** Gap junctions formed by connexins are channels on cytoplasm functioning in ion recycling and homeostasis. Some members of connexin family including connexin 31 are significant components in human skin and cochlea. In clinic, mutations of connexin 31 have been revealed as the cause of a rare hereditary skin disease called erythrokeratodermia variabilis (EKV) and non-syndromic hearing loss (NSHL).

**Objective:** To determine the underlying genetic cause of EKV, ichthyosis and NSHL in three members of a Chinese pedigree and skin histologic characteristics of the EKV patient.

**Methods:** By performing whole exome sequencing (WES), Sanger sequencing and skin biopsy, we demonstrate a Chinese pedigree carrying a mutation of *GJB3* with three patients separately diagnosed with EKV, ichthyosis and NSHL.

**Results:** The proband, a 6-year-old Chinese girl, presented with demarcated annular red-brown plaques and hyperkeratotic scaly patches on her trunk and limbs. Her mother has ichthyosis with hyperkeratosis and geographic tongue while her younger brother had NSHL since birth. Mutation analysis revealed all of them carried a heterozygous missense mutation c.293G>A of *GJB3*. Skin biopsy showed many grain cells with dyskeratosis in the granular layer. Acanthosis, papillomatosis, and a mild superficial perivascular lymphocytic infiltrate were observed.

**Conclusion:** A mutation of *GJB3* associated with EKV, ichthyosis and NSHL is reported in this case. The daughter with EKV and the son with NSHL in this Chinese family inherited the mutation from their mother with ichthyosis. The variation of clinical features may involve with genetic, epigenetic and environmental factors.

## Introduction

Gap junctions are channels or hemichannels assembled by connexins mediating cell-cell or cell-environment communication. Ions and small molecules can pass through gap junctions and guide embryonic development or pathogenic processes. Connexin 31(Cx31) coded by *GJB3* (NM_024009.3), is one important member of connexin family. Highly expressed in upper differentiating epidermis (Di et al., 2001) and cochlear (Richard et al., 2000), mutations of *GJB3* can result in different diseases including erythrokeratodermia variabilis (EKV) and non-syndromic hearing loss (NSHL) ranging from profound congenital deafness to mild, progressive hearing loss in late childhood.

EKV is a rare autosomal dominant skin disease featuring transient red patches that change over hours and days, along with fixed localized or generalized keratotic plaques. The disease is mainly caused by mutations in the *GJB3*, *GJB4*, and *GJA1* genes, all coding members of connexin (Cx) family (Ishida-Yamamoto, 2016). Clinical presentation of EKV associated with *GJB3* mutation can be variable ranging from typical keratotic lesions (Ishida-Yamamoto, 2016) to grey-brown and verrucous hyperkeratosis up to 2 cm thick (Glatz et al., 2011).

NSHL is a type of hereditary hearing loss without defects in other body parts and can be categorized as autosomal dominant, autosomal recessive, X-linked or mitochondrial mutation-related disease. Mutation of some important genes have been identified as the cause of NSHL, including *GJB2*, *GJB3*, and *GJB6*, which are all members of connexin family and generally involve with autosomal recessive or dominant hearing loss (Meena and Ayub, 2017). The Cx31 mutations lead to both recessive and dominant NSHL and severity can vary widely, from late-onset moderate deafness affecting high frequencies to congenital deafness (Liu et al., 2000).

Herein, we report a Chinese family with a missense mutation of *GJB3* associated with different clinical symptoms covering EKV, ichthyosis and NSHL.

## Materials and Methods

### Participants

**FIGURE 1:**
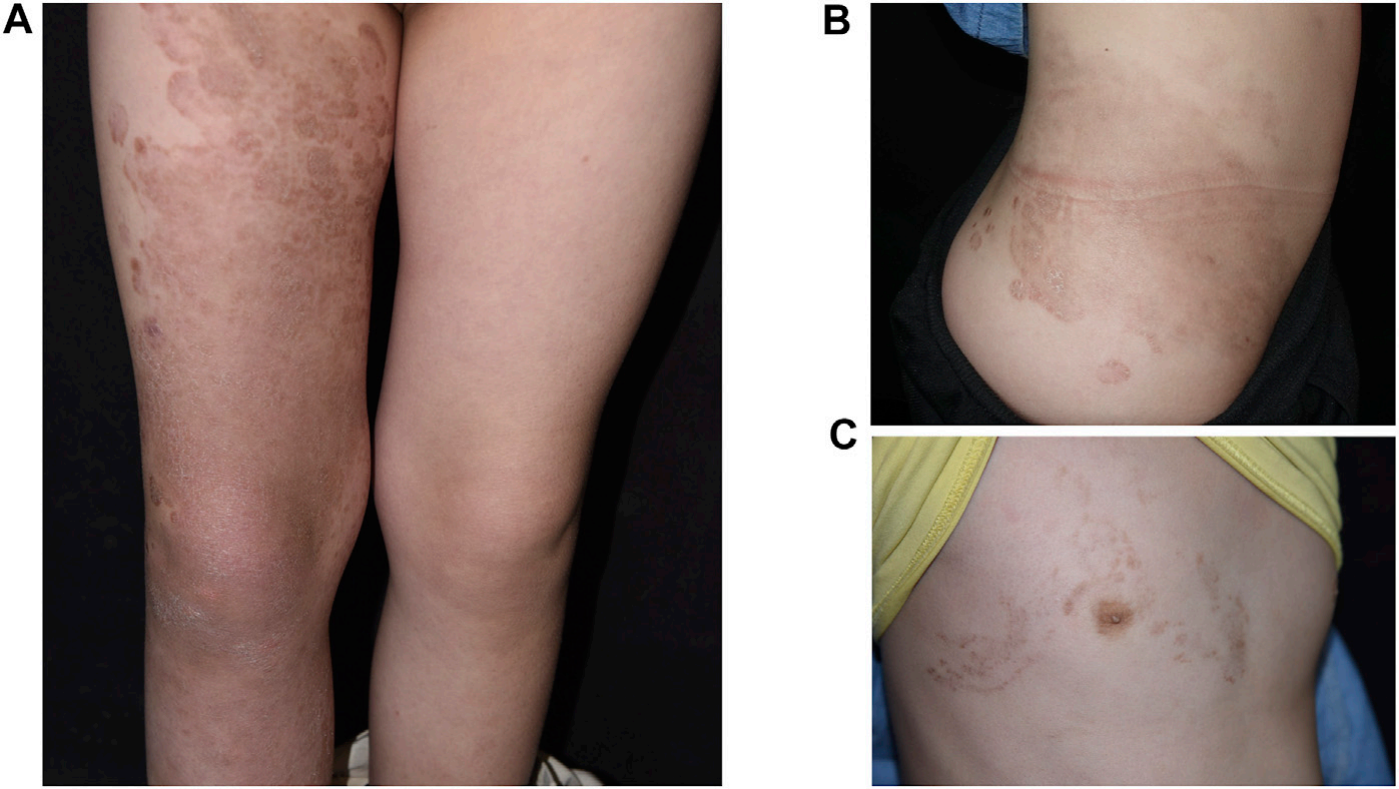
Clinical images showing demarcated, annular, red-brown plaques over the extensor side of right lower limb **(A)**, lumbar region **(B)**, and right side of the patient’s chest **(C)**.

**FIGURE 2:**
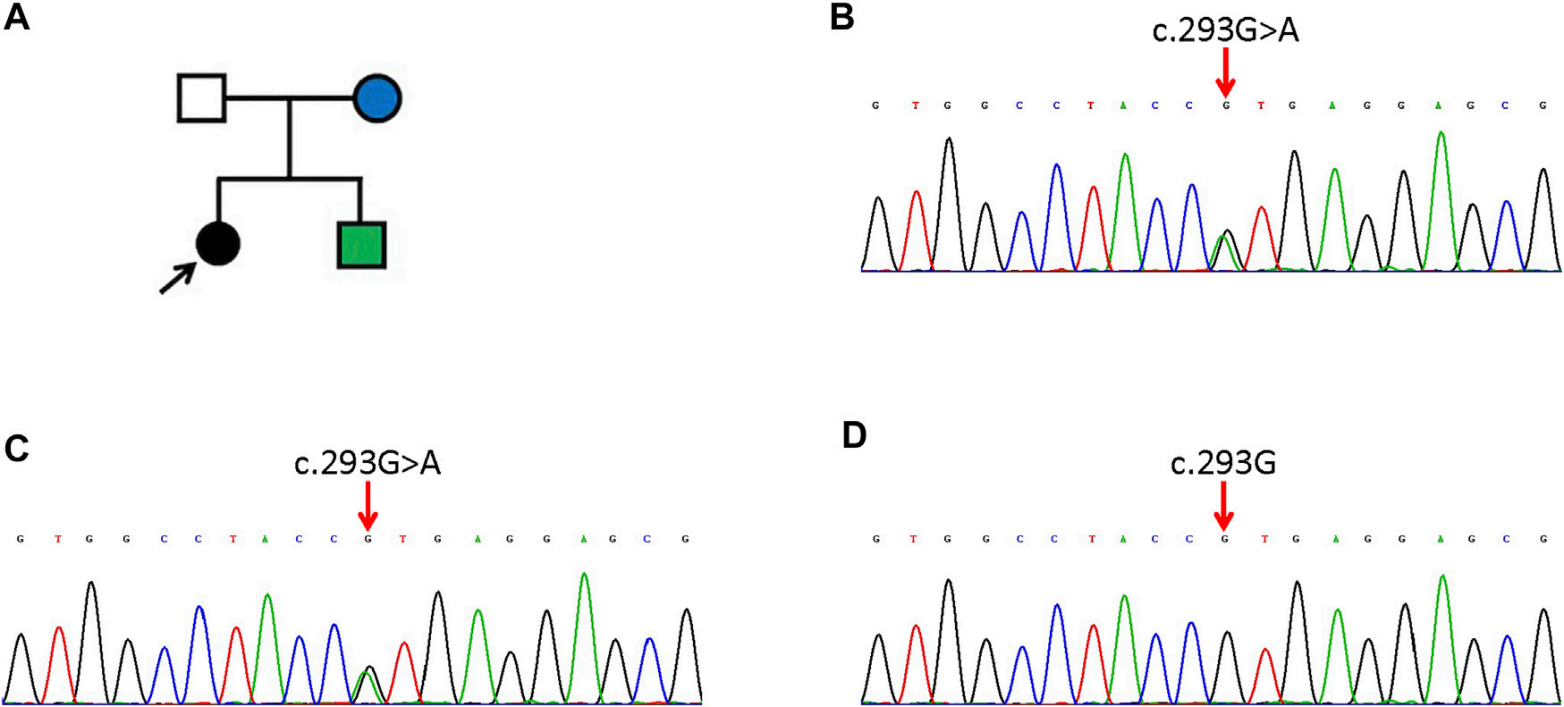
The pedigree of the family **(A)** and WES results of the proband **(B)**, her mother **(C)** and her father **(D)**. **(A)** The arrow indicates the proband. Black means EKV. The green one has NSHL and the blue one has ichthyosis. **(B,C)** The proband and her mother have a heterogenous missense mutation of c. 293G>A in *GJB3*. **(D)** The proband’s father has no mutation (c. 293G) in *GJB3*.

The study cohort includes a pair of parents, their daughter and son in a Chinese pedigree. The proband was a 6-year-old girl with demarcated annular red-brown plaques of variable sizes and colors spreading over the extensor side of right lower limb (Figure 1A), the right side of her chest (Figure 1B) and lumbar region (Figure 1C). Hyperkeratotic scaly patches were present mainly on the right thigh and knee. These manifestations had presented 6 months earlier, initially appearing on the right lower leg. No involvement of hair or nails was observed and no hearing impairment was found. Her mother has ichthyosis with hyperkeratosis on her limbs and geographic tongue while the patient’s younger brother was diagnosed with NSHL during hearing screening since birth. The father is an unaffected individual. The pedigree is shown in Figure 2A. The study was approved by institutional review board of Chinese Academy of Medical Sciences. Written informed consent was obtained from all participants, or from legal guardians in the case of minors.

### Genomic DNA Extraction

The genomic DNA was extracted from peripheral blood samples of all four participants using the QIAamp DNA Blood Mini Kit (QIAGEN, Hilden, Germany), according to the standard protocol and quantified by NanoDROP 2000 Spectrophotometer (Thermo Scientific; Waltham, MA, United States).

### Whole Exome Sequencing, Sanger Sequencing and Mutation Analysis

Whole exome sequence (WES) was conducted in the proband and her mother in Novogene company (Beijing, China) by using Illumina Novaseq plat, and the average sequencing depth is 100X. Sanger sequencing was performed in the proband’s brother for hot spot variants in NSHL-related genes (*GJB2*, *GJB3*, *SLC26A4*, and *MT-RNR1*). Raw sequence results were aligned to the human reference genome (GRCh37/hg38) annotated to get the candidate variants. Then the candidate variants were validated by Sanger sequencing to confirm the results of WES. And the primers were designed using primer3 Input for the suspected disease-causing genes.

The strategies of WES data filtering are as follows: 1) Variants with minor allele frequency (MAF)>0.01 were excluded, which were screened in normal population variant databases, including 1000G, ESP6500siv2 and gnomAD. 2) Variants in exons or alternative splicing regions were retained. 3) Synonymous mutations variants were removed, which were not located in highly conserved regions and would not affect splicing according to the same prediction software; and small non-frameshift insertion or deletion variants in the repeat regions were eliminated. 4) Variants that matched one of the following conditions were included: a) Variants were predicted to be pathogenic by at least one of the following programs including SIFT, Polyphen, MutationTaster, CADD. b) Variants were predicted to affect splicing by dbscSNV. 5) The remaining data were filtered by inheritance patterns and cutaneous phenotypes.

### Skin Biopsy

A skin biopsy was taken from the proband’s right thigh and viewed under the microscope for histopathological examination after hematoxylin-eosin staining.

## Results

### WES Result

Genetic tests revealed a highly pathogenic heterozygous missense mutation of *GJB3* in the daughter and mother (Figure 2B, C). Sanger sequencing confirmed the existence of the same mutation in the younger brother. This mutation (NM_024009.3; c.293G>A; p.R98H) resulted in a change from a highly alkaline arginine residue at codon 98 to a slightly alkaline histidine residue, between the second transmembrane helix and intracellular domain of Cx31. The mutation was not detected in the father or healthy controls (Figure 2D). A diagnosis of EKV was made for the proband.

### Histopathological Result

**FIGURE 3:**
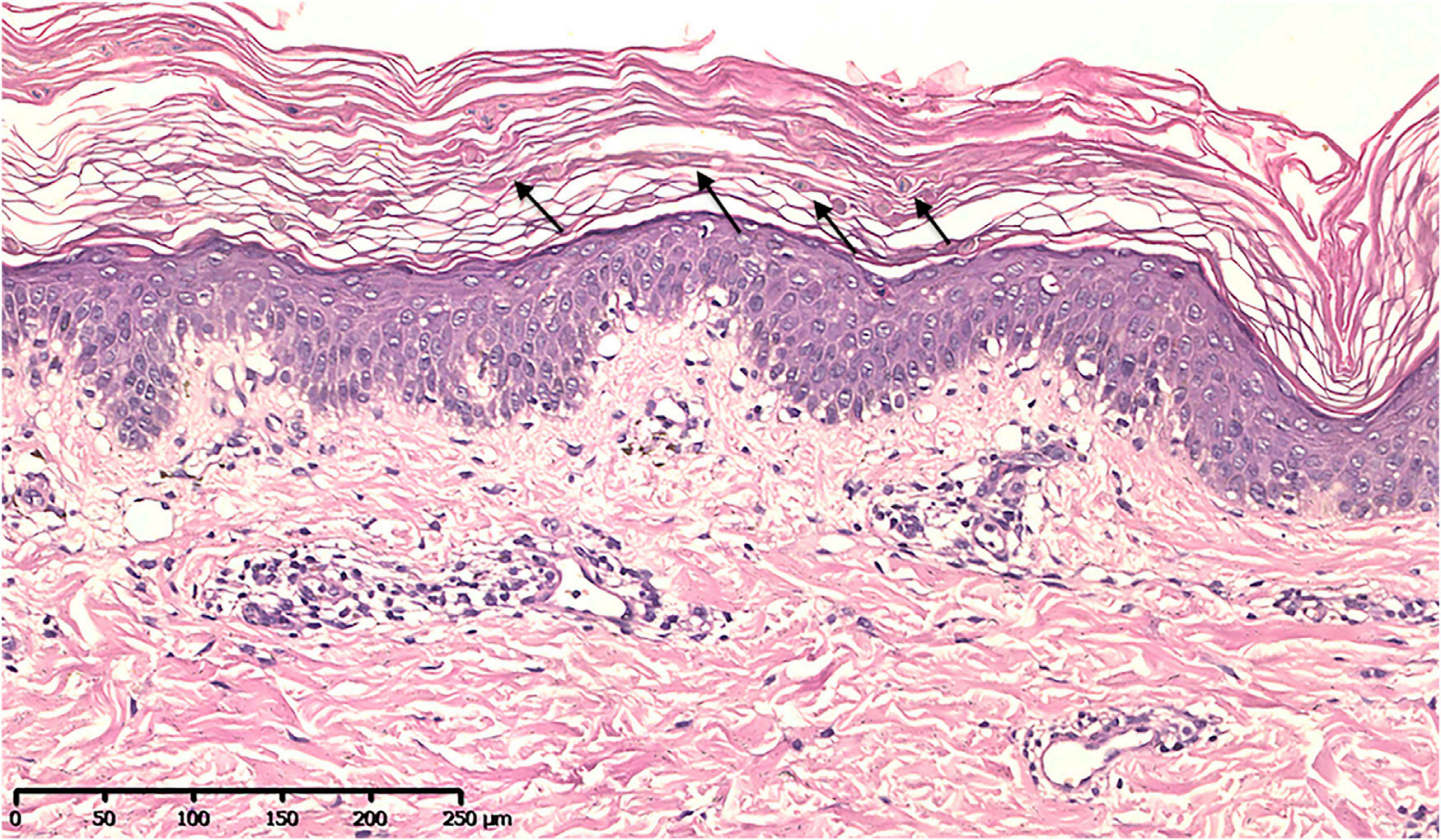
Histopathological image showing many grain cells with dyskeratosis in the granular layer, acanthosis, papillomatosis, and a mild superficial perivascular lymphocytic infiltrate (H&E).

Histopathological examination showed many grain cells with dyskeratosis in the granular layer. Acanthosis, papillomatosis, and a mild superficial perivascular lymphocytic infiltrate were observed (Figure 3).

## Discussion and Literature Review

Gap junctions are important for exchange of metabolites, ions and secondary messengers, especially in skin and cochlea. There are more than eight kinds of connexins expressed in skin epidermis, which contribute to its differentiation (Richard et al., 2000). Exchange of ions and small molecules helps maintain unique electrochemical environments which is important for cochlea normal function (Cohen-Salmon et al., 2002). *GJB3* encodes Cx31 and is highly expressed in epidermis and cochlea, forming gap junctions (Scott and Kelsell, 2011), which is important in differentiation of keratinocytes and transfer of nerve pulses (Martinez et al., 2009). Gap junctions can be homomeric (consisting of one connexin type) or heteromeric (consisting of more than one connexin type) within the same cell (Kelly et al., 2015). Therefore, the connexons formed in epidermis and cochlea are intricate and delicate to guide the differentiation and maintain normal function.

EKV is a rare autosomal dominant skin disease associated with mutation of connexin genes, including *GJB3*, *GJB4*, and *GJA1* (Ishida-Yamamoto, 2016). Several cases of autosomal recessive mutations of *GJB3* causing EKV have also been reported (Gottfried et al., 2002; Terrinoni et al., 2004; Fuchs-Telem et al., 2011; Deng et al., 2019). Transient red patches and keratotic plaques are two prominent features of EKV. In this case, the patient with EKV and her mother both carry R98H mutation in Cx31 but the mother only shows the symptom of keratotic plaques and were diagnosed with ichthyosis. A severe case of EKV with grey-brown and verrucous hyperkeratosis up to 2 cm thick was reported caused by mutation of *GJB3* (Glatz et al., 2011). Therefore, clinical symptoms of EKV may be diverse. Other genetic, epigenetic, and environmental factors are probably the explanation for variation of symptoms (Renner et al., 2008). Deep investigation is still needed. For the younger brother, no manifestation of skin is probably due to late onset characteristic of EKV or other factors related to genetics and environment.

Many kinds of connexins have been identified in cochlea and among them, Cx26 and Cx30 are predominant components while other types are limited (Wingard and Zhao, 2015). The mutations of Cx26 account for at least half of NSHL cases, while mutation of Cx31 is also a cause (Rabionet et al., 2000). Clinical symptoms of hearing loss resulted by *GJB3* mutations range from congenital hearing loss since birth to late-onset hearing loss during childhood (Wingard and Zhao, 2015). Most NSHL cases related to Cx31 mutation are autosomal recessive while a few autosomal dominant cases were also reported (Liu et al., 2012; Oh et al., 2013). However, no case carrying the Cx31 mutation with both EKV and NSHL was reported but a pedigree with both Cx26 and Cx31 mutation presented hearing loss and palmoplantar keratoderma (Kelsell et al., 2000). Therefore, one possible explanation is that other connexin protein may make up the function loss of Cx31 in skin or cochlea while more studies are still required. In this family, three people harbor the same mutation but only the son has NSHL, which is probably due to partial penetrance. In earlier reports, female carriers with *GJB3* dominant mutations in two deafness families have subclinical deafness or normal hearing while male carriers have NSHL (Xia et al., 1998), which indicates partial penetrance involving sex may be the reason of different symptoms of carriers.

**FIGURE 4:**
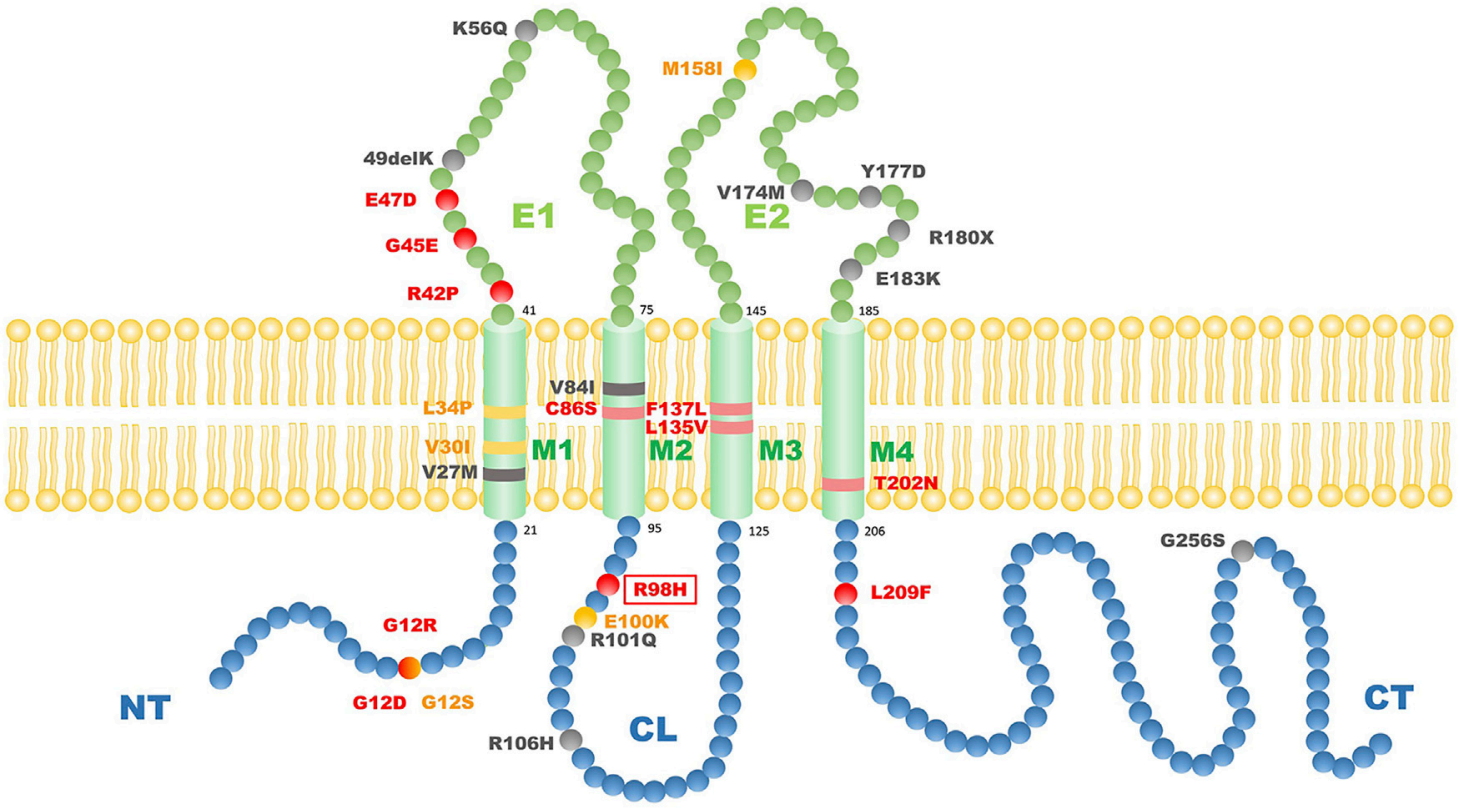
The scheme of reported GJB3 mutation related to EKV and autosomal dominant GJB3 point mutations related to NSHL. Red balls indicate autosomal dominant mutations with EKV phenotypes; yellow balls indicate autosomal recessive mutations with EKV phenotypes; black balls indicate common autosomal dominant GKB3 mutation related to NSHL. The red frame indicates the mutation we report in this case. M1–M4 refers to transmembrane domains. E1 and E2 refer to extracellular domains. CL refers to cytoplasmic loop. NT refers to cytoplasmic amino terminus. CT refers to cytoplasmic carboxy terminus.

How the mutation in Cx31 affects cell function is believed to be related to where the mutation site lies (Sugiura et al., 2015). The structure of Cx31 mainly contains four transmembrane domains (M1-4) linked by one intracellular loop (CL) and two extracellular loops (E1 and E2) with conserved cysteine residues while N- and C-termini (NT and CT) are lying inside the cell (Kelly et al., 2015; Figure 4). The E1 domain plays an important role in formation of the gap junction channel (Richard et al., 2000). The M2 domain is known for function in voltage gating (Rabionet et al., 2000). The extracellular domain E2 probably functions in interaction between different types of connexin and formation of heterotypic connexons (Sugiura et al., 2015). Mutations of *GJB3* resulting in NSHL mainly locate in E2 domain, which may interfere the interaction between Cx31 and Cx26 and damage the function of heterotypic connexons on the membrane of cochlear cells (Sugiura et al., 2015). However, there is seemingly no relationship between the mutation locus and phenotypes of EKV patients. Most mutations related to EKV are autosomal dominant while a few recessive mutations were also found (shown in Figure 4). Interestingly, a compound heterozygous case with two recessive mutations in *GJB3* presented a mutation lying in E2, which was the first pathologic mutation involved with EKV identified in this domain (Deng et al., 2019). This patient had no symptoms of hearing loss probably because this mutation in E2 domain is recessive. By systematically searching the PubMed, Embase and Web of Science, we summarized all the *GJB3* mutations reported leading to EKV and phenotypes in each case (Table 1) and autosomal dominant *GJB3* mutation related to NSHL (Figure 4; Richard et al., 1998; Xia et al., 1998; Wilgoss et al., 1999; Lopez-Bigas et al., 2000; Richard et al., 2000; Gottfried et al., 2002; Alexandrino et al., 2004; Terrinoni et al., 2004; Common et al., 2005; Feldmeyer et al., 2005; Morley et al., 2005; Yang et al., 2007; Renner et al., 2008; Li et al., 2010; Fuchs-Telem et al., 2011; Glatz et al., 2011; Scott and Kelsell, 2011; Wang et al., 2011; Liu et al., 2012; Torres et al., 2012; Wang et al., 2012; Ikeya et al., 2013; Oh et al., 2013; Otaguchi et al., 2014; Beck et al., 2015; Sugiura et al., 2015; Takeichi et al., 2016; Deng et al., 2018; Imura et al., 2020). In this case, the substitution of R98H lying in the border of M2 and CL, which are important in voltage and pH gating (Richard et al., 2000), is the first mutation found involving both EKV and NSHL. The exact mechanism behind needs more investigation.

**TABLE 1:** Reported pathogenic mutations in *GJB3* related to EKV and phenotypes.

No	Hereditary mode	Erythematous plaques distribution	Palmoplantar keratoderma	Nucleotide change	Amino acid change	Protein domain	Mutation type	Novel or reference
1	AD	/	/	c.34G>C	P. G12R	NT	Missense	(Richard et al.,1998)
2	AD	/	/	c.35G>A	p. G12D	NT	Missense	(Richard et al.,1998)
3	AR	Face, limbs, buttocks, and chest	Y	c. 34G>A	p. G12S	NT	Missense	(Deng et al., 2018)
4	AR	Back	Y	c.88G>A	p. V30I	M1	Missense	(Fuchs-Telem et al, 2011)
5	AR	Abdomen, trunk, earlobes and extensor aspects of the upper and lower limbs	N	c. 101T>C	p. L34P	M1	Missense	(Gottfried et al., 2002)
6	AD	① /	① Y	c.125G>C	p. R42P	E1	Missense	① (Richard et al., 2000)
		② Buttocks, lower back, neck and four limbs	② Y					② (Wilgoss et al., 1999)
7	AD	① Whole body	Y	c.134G>A	p. G45E	E1	Missense	① (Wang et al., 2012)
		② The extensor sides of the extremities and the face						② (Renner et al., 2008)
8	AD	Body and limbs	Y	c. 141G>C	p. E47D	E1	Missense	(Wang et al., 2011)
9	AD	① /	/	c.256T>A	p. C86S	M2	Missense	(Richard et al.,1998)
10	AD	right side of chest, waist, and extensor side of right leg	N	c.293G>A	p. R98H	CL	Missense	Novel
11	AR	Whole body	Y	c. 829G>A	p. E100K	CL	Missense	(Terrinoni et al., 2004)
12	AD	Trunk and limbs	/	c. 403C>G	p. L135V	M3	Missense	(Scott et al., 2011)
13	AD	① Four extremities	① Y	c. 409 T>C	p. F137L	M3	Missense	① (Richard et al.,2000)
		② Back and four limbs	② /					② (Glatz et al., 2011)
		③ Face, upper trunk, arms, and buttocks	③ Y					③ (Imura et al., 2020)
14	AR	Face, limbs, buttocks, and chest	Y	c. 474G>A	p. M158I	E2	Missense	(Deng et al., 2018)
15	AD	Trunk and the extremities	Y	c. 605C>A	p. T202N	M4	Missense	(Sugiura et al., 2015)
16	AD	① Forehead, cheeks, extremities and buttocks	① Y	c. 625C>T	p. L209F	CT	Missense	① (Morley et al., 2005)
		② Back and limbs	② Y					② (Otaguchi et al., 2014)
		③ Extensor surfaces and buttocks; buttocks, trunk, face and extremities and extensor surfaces; limbs and buttocks; buttocks and right arm.	③ Y in 2 women and 1 man, N in 1 man					③ (Feldmeyer et al., 2005)

AD, autosomal dominant; AR, autosomal recessive; Y, yes; N, no.

Although the phenotypes of different pathologic mutations may be the same, the mechanisms behind them are likely different. In many *vitro*-studies, overexpression of Cx31 with the same mutation in cells may obtain different conclusions about pathogenic mechanisms possibly due to different experimental conditions. But overall, the viability of cells with EKV-related mutated Cx31 was decreased, while that of cells with NSHL-related Cx31 mutation was not (He et al., 2005; Tattersall et al., 2009; Easton et al., 2019). The mechanisms behind can be concluded into mainly two ways: 1) The mutated Cx31 protein accumulates in endoplasmic reticulum (ER) due to misfold, leading to ER stress response and finally cell death (Di et al., 2002; Tattersall et al., 2009; Chi et al., 2012). 2) Mutated Cx31 can be transferred to the cell membrane but only form dysfunctional gap junctions which may even interfere the normal function of plasma membrane (Rouan et al., 2003). However, a kind of rare mutation of Cx31 with G45E exhibits a new way to damage cells by inducing necrosis (Easton et al., 2019). Overexpression of Cx31G45E-GFP within HeLa cells and HaCaT cells led to expansion of the ER due to accumulation of mutated protein and finally cell necrosis rather than ER stress responses (Easton et al., 2019). Also, the interaction between mutated Cx31 and other wild-type connexins enables the accumulation of normal connexin in ER, which decreases the gap junctions on the cell membrane and interferes with normal function (Easton et al., 2019). The pathogenic mechanism of R98H in Cx31 needs experiments *in vitro* to identified.

In this case, we report a Chinese family with a mutation associated with EKV, ichthyosis and NSHL. The daughter with EKV and the son with NSHL in this Chinese family inherited the mutation from their mother with ichthyosis. The variation in clinical features may involve with genetic, epigenetic and environmental factors. One shortage of our research is that further experiments *in vitro* are needed to identify the possible pathogenic mechanism of this mutation. Our results indicate an important mutation site of Cx31 leading to EKV and NSHL with partial penetrance.

## Data Availability

The datasets presented in this study can be found in online repositories. The names of the repository/repositories and accession number(s) can be found below: GenBank database, accession number OL471368.
